# Supplementary material for: Intent to Vaccinate SARS-CoV-2 Infected Children in US Households: A Survey
Source: Vaccines (Basel). 2021 Sep 21;9(9):1049. doi: 10.3390/vaccines9091049 (PMC8473386; doi:10.3390/vaccines9091049)
Supplement: Supplementary file 1 [file vaccines-09-01049-s001.zip › vaccines-1340772-supplementary.pdf]

Supplemental Table S1: Survey questions

1. If a safe and effective vaccine against SARS-CoV-2 becomes available, would you allow it to be given to your child?
   a. Yes
   b. No
   c. Maybe/undecided
      i. If no/maybe, what are your concerns?
2. If a vaccine against SARS-CoV-2 becomes available, would you allow it to be given to other members in your home including yourself?
   a. Yes
   b. No
   c. Maybe/undecided
      i. If no/maybe, what are your concerns?
3. If your child is vaccinated, do you believe they will help prevent others from becoming infected?
   a. Yes
   b. No
   c. Maybe
      i. Vaccination efficacy notes:
4. *What information have you heard or read about COVID-19? [open ended]
5. *Where did you read/hear about a COVID-19 vaccine [newspaper/online, social media, other]. The response "other" triggered further explanation.
6. *Was the report pro-vaccine, anti-vaccine or both?
7. *If a vaccine prevents 19 of 20 people against getting COVID-19 disease, would you allow it to be given to your children, including the child who received care.

*Question was only asked on secondary survey

Supplemental Table S2: Comparison of first and second survey responses

**Characteristic**	N = 45[1]
**Whether would vaccinate child**	
Maybe to maybe	3 / 45 (6.7%)
Maybe to no	1 / 45 (2.2%)
Maybe to yes	1 / 45 (2.2%)
No to maybe	3 / 45 (6.7%)
No to no	8 / 45 (18%)
No to yes	4 / 45 (8.9%)
Yes to maybe	4 / 45 (8.9%)
Yes to no	5 / 45 (11%)
Yes to yes	16 / 45 (36%)
**Whether would vaccinate other household members, including self**	
Maybe to maybe	2 / 45 (4.4%)
Maybe to no	1 / 45 (2.2%)
Maybe to yes	1 / 45 (2.2%)
No to maybe	5 / 45 (11%)
No to no	8 / 45 (18%)

No to yes	4 / 45 (8.9%)
Yes to maybe	5 / 45 (11%)
Yes to no	3 / 45 (6.7%)
Yes to yes	16 / 45 (36%)
**Whether vaccinating the child would prevent others from infection**	N = 44[1*]
Maybe to maybe	3 / 44 (6.8%)
Maybe to no	1 / 44 (2.3%)
Maybe to yes	1 / 44 (2.3%)
No to maybe	3 / 44 (6.8%)
No to no	3 / 44 (6.8%)
No to yes	9 / 44 (20%)
Yes to maybe	3 / 44 (6.8%)
Yes to no	1 / 44 (2.3%)
Yes to yes	20 / 44 (45%)

[1] Statistics presented: n / N (%)

*1 participant did not answer this question
